# A Missense Mutation in Close Proximity of ALS-linked *PFN1* Mutations Causes Only Early-onset Paget Disease of Bone

**DOI:** 10.1210/clinem/dgaf314

**Published:** 2025-06-03

**Authors:** Rou Weng, Xiaoxiang Li, Hua Yue, Yang Xu, Zhe Wei, Shuqin Xu, Baojie Li, Zhenlin Zhang

**Affiliations:** Department of Osteoporosis and Bone Disease, Shanghai Clinical Research Center of Bone Disease, Sixth People's Hospital, Shanghai Jiao Tong University School of Medicine, Shanghai 200233, China; Department of Orthopedics Surgery, Tangdu Hospital, The Fourth Military Medical University, Xi’an, Shaanxi 710038, China; Department of Osteoporosis and Bone Disease, Shanghai Clinical Research Center of Bone Disease, Sixth People's Hospital, Shanghai Jiao Tong University School of Medicine, Shanghai 200233, China; Department of Osteoporosis and Bone Disease, Shanghai Clinical Research Center of Bone Disease, Sixth People's Hospital, Shanghai Jiao Tong University School of Medicine, Shanghai 200233, China; Department of Osteoporosis and Bone Disease, Shanghai Clinical Research Center of Bone Disease, Sixth People's Hospital, Shanghai Jiao Tong University School of Medicine, Shanghai 200233, China; Department of Osteoporosis and Bone Disease, Shanghai Clinical Research Center of Bone Disease, Sixth People's Hospital, Shanghai Jiao Tong University School of Medicine, Shanghai 200233, China; Bio-X Institutes, Key Laboratory for the Genetics of Developmental and Neuropsychiatric Disorders, Ministry of Education, Shanghai Jiao Tong University, Shanghai 200240, China; Department of Osteoporosis and Bone Disease, Shanghai Clinical Research Center of Bone Disease, Sixth People's Hospital, Shanghai Jiao Tong University School of Medicine, Shanghai 200233, China; Bio-X Institutes, Key Laboratory for the Genetics of Developmental and Neuropsychiatric Disorders, Ministry of Education, Shanghai Jiao Tong University, Shanghai 200240, China; Department of Osteoporosis and Bone Disease, Shanghai Clinical Research Center of Bone Disease, Sixth People's Hospital, Shanghai Jiao Tong University School of Medicine, Shanghai 200233, China

**Keywords:** Paget's disease of bone, genetic animal model, osteoclasts

## Abstract

**Context:**

Paget disease of bone (PDB) is a metabolic disorder characterized by abnormal osteoclast activation. Recently, mutations in the *PFN1* gene, which encodes Profilin 1, an actin-binding protein controlling actin dynamics and cell movement, have been linked to early-onset PDB. Interestingly, mutations in *PFN1* (C71G, T109M, M114T, E117G, G118V, etc.) are associated with amyotrophic lateral sclerosis (ALS), a neurodegenerative disorder affecting motor neurons.

**Objective:**

To provide insights into the underlying molecular mechanism of early-onset PDB.

**Methods:**

We observed the clinical responses to denosumab in early-onset PDB patients. Additionally, a mouse model carrying the c.335T>C mutation in the *Pfn1* gene was generated.

**Results:**

We reported a second Chinese family affected by early-onset PDB with malignant giant cell tumors, in which we identified the same heterozygous missense mutation (c.335T>C/p. L112P) in *PFN1* that we have reported previously in another family. Despite its proximity to ALS-linked *PFN1* mutations, the *PFN1* L112P mutation did not induce ALS in affected individuals. These early-onset PDB patients exhibited a significantly poorer response to denosumab compared to typical PDB patients. The heterozygous mice displayed PDB-like phenotypes, including skeletal deformities and focal osteoclastic lesions with giant osteoclasts, and did not show ALS-like phenotypes. We further show that mutation of *Pfn1* leads to enhanced actin ring-like structures at the bone surfaces without affecting nuclear factor-κB activation in osteoclast cultures.

**Conclusion:**

The observation of recurrent mutations highlights the causative role of *PFN1* (L112P) in early-onset PDB/giant cell tumor within the Chinese population and provides insights into the physio-pathological functions of Profilin 1.

Paget disease of bone (PDB [MIM: 167250]) is a metabolic bone disorder with significant genetic background. It is characterized by bone pain, enlargement, deformation of 1 (monostotic form) or more (polyostotic form) bones, and pathological fractures (1). The PDB pathogenesis involves abnormal activation of osteoclasts, leading to compensatory bone formation mediated by osteoblasts. This dysregulated bone remodeling primarily affects the pelvis, spine, femur, skull, and tibia (2). The most severe complication of PDB is the neoplastic degeneration of affected bone containing osteosarcoma, fibrosarcoma, chondrosarcoma, or giant cell tumor (GCT) (3-6). The majority of PDB with GCT patients have a positive family history, elevated levels of serum alkaline phosphatase (ALP), and the mortality rate was much higher than PDB patients without GCT (approximately 50% vs 0 to 5% at 5 years) (5).

Approximately 5% to 40% of patients diagnosed with PDB have a positive family history, highlighting the substantial influence of genetic factors in PDB or PDB/GCT etiology (7, 8). Inheritance of the disease typically follows an autosomal dominant pattern with incomplete penetrance. Currently, mutations in the genes, including *SQSTM1* [MIM: 601530] (9), *TNFRSF11A* [MIM: 603499] (10), *OPG [239000]*, *VCP* [MIM: 601023] (11), *ZNF687* [MIM: 610568] (12)*, HNRNPA1* [MIM: 164017] (13), and *HNRNPA2B1* [MIM: 600124] (14) are associated with PDB or PDB-related syndromes. However, the underlying molecular mechanisms of PDB, particularly in cases involving PDB/GCT, are not fully understood yet.

In 2020, a frameshift mutation (D107Rfs*3) in the Profilin 1 (*PFN1*) gene was identified as a genetic factor associated with early-onset PDB in 2 large Italian pedigrees (15, 16). Subsequently, we identified the same *PFN1* frameshift mutation (D107Rfs*3) in a large Chinese pedigree with PDB/GCT in 2021 (17). Moreover, we revealed a heterozygous 1-bp deletion in the *PFN1* (c.324_324delG) gene in patients with sporadic early-onset PDB/GCT and a heterozygous missense mutation in *PFN1* (L112P) gene in another family with PDB/GCT (17). These findings suggested that both deletions and missense mutations in *PFN1* might contribute to the development of PDB/GCT.

PFN1, a highly conserved protein weighing 15 kD, is expressed ubiquitously across various cell types and is crucial for regulating actin cytoskeleton turnover and restructuring (18, 19). It possesses an actin-binding domain located within helix 3 and a poly-L-proline-binding domain at the N-terminal (20, 21). Previously, mutations in the *PFN1* gene, particularly missense mutations, were identified in patients with amyotrophic lateral sclerosis (ALS), a late-onset neurodegenerative disorder caused by the death of motor neurons (22, 23). Patients with ALS typically face paralysis and eventual respiratory failure within 3 to 5 years of diagnosis (24). Using exome sequencing, 8 mutations, including A20T, C71G, T109M, M114T, E117G, G118V, R136W, and Q139L, in the *PFN1* gene were identified in patients with familial and sporadic ALS (23, 25-27). Notably, the T109M, M114T, E117G, and G118V mutations are located in or near the last β-sheet of PFN1. Recent studies demonstrated that *PFN1* mutations triggered the accumulation of TDP-43 in motor neurons, a coactivator of the nuclear factor-κB (NF-κB) P65 subunit, enhancing the activation of NF-κB signaling (28, 29). Recent investigations also support the notion that *PFN1* plays a negative role in NF-κB activation in human trophoblast cells and human breast cancer cells (30, 31). Additionally, ALS-*PFN1* variants might affect microglial vesicular degradation by binding to phosphatidylinositol (4,5)-bisphosphate (32).

The current study reported early onset of PDB in 2 individuals of another Chinese family. Among them, 1 also developed GCT. Notably, despite carrying a *PFN1* (L112P) mutation, these patients did not manifest ALS phenotypes. The current study further revealed their limited response to zoledronic acid or denosumab treatment compared to typical PDB patients. Moreover, by generating *Pfn1* (L112P) knock-in mice, it was observed that the heterozygous mutant mice exhibited PDB phenotypes without displaying ALS-related symptoms. Moreover, the mutation resulted in an increased number of osteoclasts and the formation of giant osteoclasts in vitro and in vivo. Mechanistically, heterozygous mutation of *Pfn1* leads to enhanced actin ring-like structures at the bone surfaces without affecting NF-κB activation in osteoclast cultures. These findings demonstrated the pathogenic role of the *PFN1* (L112P) mutation in early-onset PDB. Despite its close proximity to ALS-linked *PFN*1 mutations, the *PFN1* (L112P) mutation only causes PDB but not ALS, thereby emphasizing the highly regulated and multifaceted functions of Profilin 1 in cellular processes.

## Materials and Methods

### Subjects

The current study involved 2 Chinese Han pedigrees with early-onset PDB/GCT. Family 1 was from Shaanxi Province (Northwest China) and was diagnosed and treated at the Department of Orthopedics Surgery, Fourth Military Medical University-affiliated Tangdu Hospital, and Department of Osteoporosis and Bone Disease, Shanghai Jiao Tong University of Medicine-Affiliated Sixth People's Hospital, respectively. Family 2 was from Jiangsu Province (East China) and recruited at Shanghai Jiao Tong University Affiliated Sixth People's Hospital, as reported in our previous study (17). Moreover, 500 healthy volunteers of Han Chinese descent served as controls. The ethical approval for the study was obtained from the Ethics Committee of Shanghai Jiao Tong University Affiliated Sixth People's Hospital. All participants provided informed consent before participating.

### DNA Isolation and Sanger Sequencing

The proband provided peripheral blood samples after giving informed consent. Genomic DNA extraction was carried out using the QuickGene DNA Whole-Blood Kit and the Nucleic Acid Isolation System QuickGene-610 L (Fujifilm, Tokyo, Japan), following the manufacturer's instructions. The PCR amplification of all exons and introns of the *PFN1* gene was performed using the proband's genomic DNA to identify mutations. The primer sequences were designed using Primer3 (v0.4.0).

### Bioinformatics Analysis

The deleterious impacts of missense mutation (L112P) were evaluated using online databases, including Polymorphism Phenotyping v2, Protein Variation Effect Analyzer, and MutationTaster.

### Generation of *Pfn1*^L112P^ Mouse Model

The current study employed the principle of homologous recombination and used embryonic stem (ES) cell targeting to induce a point mutation in the *Pfn1* gene in mice. Briefly, the ES cell targeting vector was constructed using the infusion method, consisting of a 3.2 kb 5′ homologous arm, PGK-Neo-polyA, 2.9 kb 3′ homologous arm, and MC1-TK-polyA as a negative selection marker. Following linearization, the vector was incorporated into JM8A3 ES cells using electroporation. Subsequently, a correct homologous recombined positive clone was identified using long-range PCR. The chimeric mice were generated by injecting positive ES cells into the embryos of C57BL/6J mice. These chimeric mice were then allowed to mate with Flp mice to generate positive F1 generation mice with Neo-deleted heterozygosity. The colony was maintained by breeding heterozygotes with both wild-type (WT) and heterozygous littermate (*Pfn1*^L112P^) animals used in the current study.

### Micro-CT Analysis

The femurs of the 4-month-old mice were dissected to remove most soft tissue and then fixed in 4% paraformaldehyde (PFA) for 24 hours, followed by preservation in 75% ethanol. Bone morphometry assessment was conducted by scanning the left femur at a resolution of 9 μm using a Skyscan 1176 system (Bruker, Kontich, Belgium). The reconstruction and analysis of trabecular bone parameters, including the volumetric bone mineral density, bone volume fraction (bone volume/total volume), trabecular thickness, trabecular separation, trabecular number, and cortical thickness, were performed using Skyscan, NRecon, Data Viewer, CTAn, CTvox, and Batman software.

## X-Ray Imaging

The 8-week-old mice were injected with anesthetic into the abdominal cavity, followed by an evaluation of bone morphology in the skull, spine, femur, and tibia using X-ray imaging.

### Bone Histology, Immunohistochemistry and Immunofluorescence Staining

Mice were euthanized by air embolism, and the femurs and skulls were fixed in 4% PFA at room temperature (RT) for 24 hours. Paraffin-embedded decalcified sections were cut into 6-µm-thick sections and subsequently stained with hematoxylin and eosin. The osteoclasts were visualized by staining the sections with a substrate specific to tartrate-resistant acid phosphatase (TRAP) using TRAP Staining Kit (Sigma-Aldrich, St. Louis, MO, USA), and incubated at 37 °C for 30 minutes. The immunohistochemistry staining involved dewaxing the sections in xylene, rehydration in alcohol, and treatment with 3% hydrogen peroxide for 20 minutes to neutralize endogenous peroxidase activity. Subsequently, the sections were placed in an acidic sodium citrate solution and fixed at 85 °C in a water bath for 20 minutes. The nonspecific binding was then blocked by incubating the sections at RT with 5% BSA in PBS for 1 hour. Then, the overnight incubation at 4 °C with rabbit anti-profilin 1 antibodies (1:100; Abcam Cat# ab124904, RRID: AB_10975882) was followed by washing 3 times in PBS with 0.1% Tween 20 and staining with PV-9000 2-Step Plus Poly-HRP Anti-Rabbit IgG Detection System (ZSGBBIO, Beijing, China) and a Liquid DAB Substrate Kit (Invitrogen, Carlsbad, CA, USA) following the manufacturer's protocol. Finally, sections were sealed with neutral resin and observed and photographed under a microscope. For immunofluorescence, sections were incubated with rabbit anti-β actin antibodies (1:100; Cell Signaling Technology Cat# 93473, RRID: AB_3099713) overnight at 4 °C. Then the sections were washed 3 times in PBS for 5 minutes each and mounted with Vectashield mounting medium containing 4′,6-diamidino-2-phenylindole (Vector Laboratories) and sealed with nail polish.

### Cell Culture and Differentiation

Bone marrow cells were extracted from the hindlimbs of WT and *Pfn1*^L112P^ mice. These cells were cultured in α-MEM supplemented with 15% fetal bovine serum and 1% penicillin/streptomycin in 60-mm cell culture dishes. Following overnight incubation at 37 °C with 5% CO_2_, the nonadherent bone marrow cells were transferred to 12-well plates at a density of 1 × 10^6^ cells/well. The cells were then stimulated with 50 ng/mL macrophage colony-stimulating factor (Peprotech) and 100 ng/mL RANKL (Peprotech) to induce osteoclast differentiation. The culture medium was refreshed every 2 days during the 5-day observation for osteoclastic differentiation. For osteoblast differentiation, bone marrow cells was induced with 50 μg/mL ascorbic acid and 10 mM β-glycerophosphate in complete cell culture medium, with medium renewal every 3 days. Following 7 days of osteogenic differentiation, cells were fixed in 4% PFA for 10 minutes. ALP staining was carried out using the NBT/BCIP staining kit according to the manufacturer's instructions. Mineralized nodule formation was determined using Alizarin Red Staining (Beyotime, C0148S) after osteogenic incubation for 21 days, as stated in the manufacturer's instructions.

### Resorption Pit Staining

A resorption pit formation assay was performed using bone slices. Bone slices were placed in 96-well plates, and bone marrow cells were seeded and differentiated according to previously outlined procedure. On the 21st day, the slices were fixed with 2.5% glutaraldehyde and dehydrated through a series of ethanol gradients. Subsequently, the resorption pits on the bone slices were visualized and captured via scanning electron microscopy after gold coating.

### TRAP Staining

Bone marrow cells were cultured and subjected to differentiation according to the previous described protocol. Following differentiation, the cells were fixed in 4% PFA, followed by TRAP staining using a TRAP Staining Kit from Sigma-Aldrich. Mature osteoclasts were identified as TRAP-positive cells with more than 3 nuclei. The quantification of mature osteoclasts was performed using Image J software.

### Quantitative Real-Time PCR

The total RNA was extracted from osteoclasts using Trizol reagent (Invitrogen, Carlsbad, CA, USA). cDNA was synthesized using HiScript III RT SuperMix for quantitative PCR (qPCR) (+gDNA wiper) (Vazyme, Nanjing, China). For qRT-PCR assays, SYBR Premix Ex Taq (TaKaRa, Dalian, China) was used. The relative expressions of mRNAs were normalized to β-actin using the 2^−ΔΔCt^ method. The following primer sequences were used for amplification: *c-Fos* forward 5′-TGTTC CTGGC AATAG CGTGT-3′ and reverse 5′-TCAGA CCACC TCGAC AATGC-3′; *β-actin* forward 5′-TCCTT CTTGG GTATG GAA-3′ and reverse 5′-AGGAG GAGCA ATGAT CTTGA TCTT-3′; *TRAP* forward 5′ CTGGA GTGCA CGATG CCAGC GACA-3′ and reverse 5′AGACC AGGTA GCGGC TCGTG CCT3′; *COL1* forward 5′-TGGAA GAGTG GAGAG TACTG GAT-3′ and reverse 5′-ATACT CGAAC TGGAA TCCAT CGG −3′; *ALP* forward 5′-TCAGG GCAAT GAGGT CACAT C-3′ and reverse 5′-CACAA TGCCC ACGGA CTTC-3′; and *RUNX2* forward 5′-CGGCC CTCCC TGAAC TCT-3′ and reverse 5′-TGCCT GCCTG GGATC TGTA-3′.

### Western Blot

Total protein was extracted from the osteoclast differentiated from bone marrow cells of *Pfn1*^L112P^ mice and their WT counterparts. Equal amount of protein samples were loaded onto SDS-polyacrylamide gels for separation by electrophoresis and subsequently transferred to polyvinylidene fluoride membranes according to standard procedures. The membranes blocked with 5% BSA and followed by washing 3 times in Tris-buffered saline with Tween. After overnight incubation at 4 °C with primary antibodies (rabbit anti-P65, 1:1000, Cell Signaling Technology Cat# 8242, RRID:AB_10859369; rabbit anti-p-P65, 1:1000, Cell Signaling Technology Cat# 3033, RRID:AB_331284; rabbit anti-Profilin 1, 1:1000, Abcam Cat# ab124904, RRID: AB_10975882; rabbit anti-β-tubulin, 1:1000, Cell Signaling Technology Cat# 15115, RRID:AB_2798712), the membranes were washed in Tris-buffered saline with Tween 3 times and incubated with horseradish peroxidase-conjugated anti-rabbit secondary antibodies. The intensity of the protein bands was quantified by Image J software.

### Rotarod Test

The rotarod experiments were commenced when the mice reached 12 weeks of age, with weekly measurements conducted for 3 consecutive weeks. The *Pfn1*^L112P^ mice and their counterparts were acclimatized with the setup for a preexperimental period of 5 days. During the experiment, mice were positioned on a stationary rotarod, which was initiated at 1 r/minute and gradually increased to 30 r/minute within 3 minutes. The duration for which each mouse remained on the rotarod was recorded. Each mouse underwent 3 trials with a 30-minute interval between each trial, and the longest duration of retention was recorded. The rotarod fatigue tester (YLS-4C) was procured from Shandong Medical Technology Co. Ltd.

### Statistical Analysis

The statistical analyses were performed using GraphPad Prism (La Jolla, CA, USA) and SPSS (SPSS Inc., Chicago, IL, USA) software. The 1-way ANOVA was employed to analyze quantitative variables from 3 or more independent groups, whereas the Student *t*-test was used for comparisons between 2 groups. A *P* value < .05 was considered statistically significant.

## Results

### PFN1 L112P Mutation is Associated With PDB/GCT Rather Than ALS in Human

**Figure 1.**
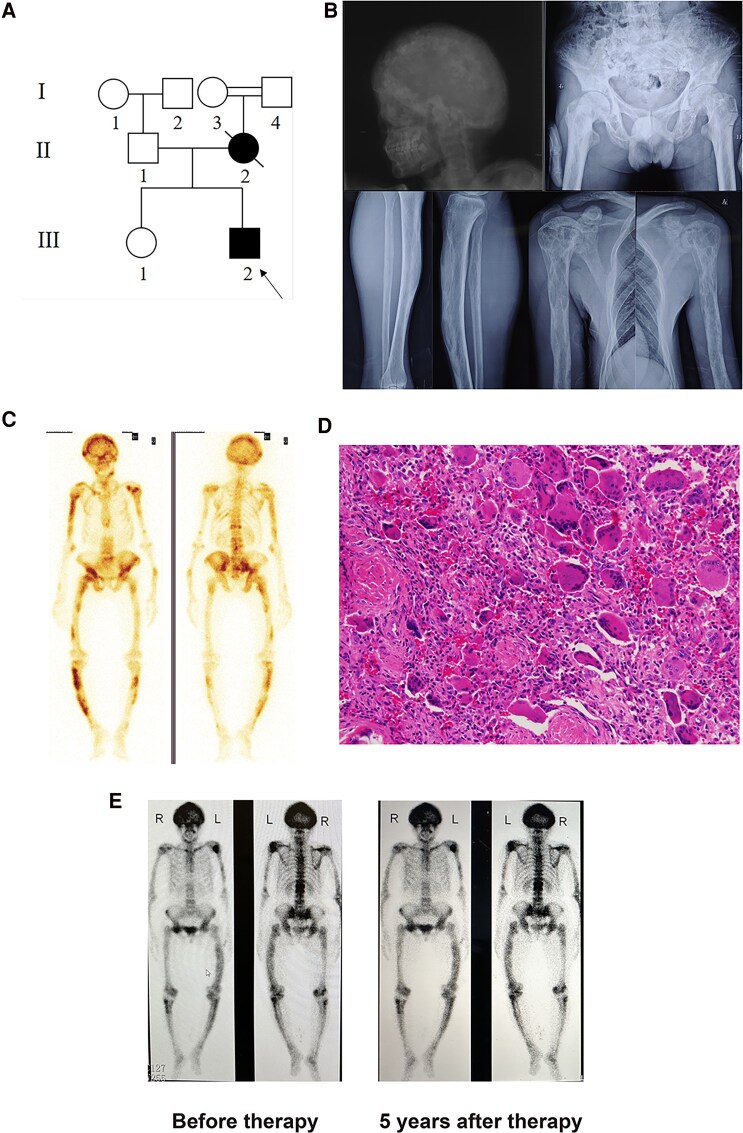
Clinical features and pedigree of family 1 and long-term effects of denosumab treatment of family 2. (A) The pedigree of family 1 is shown. The family members with Paget disease of bone (PDB) are indicated with black symbols. The black arrow indicates the proband. (B) X-ray examination of the proband showed that the deformity of femurs, pelvis, and tibias was more obvious with large and disordered trabecular bone and osteoporosis with osteolysis sites. X-ray examination of the skull, clavicles, and humeri is shown. The skull was deformed and the skull plate barrier was thickened and enlarged with sclerotic foci. (C) Whole-body bone scintigraphy of the proband showed radionuclide uptake in the skull, clavicles, spine, pelvis, humeri, ulnas, femurs, and tibias. (D) Pathological image of tumors resected from the right parietal region of the proband's head (hematoxylin and eosin staining 200×) showed numerous multinucleated giant cells. (E) Whole-body bone scintigraphy at baseline and 5 years from the beginning of denosumab (Xgeva 120 mg) treatment for patient Ⅱ.2 shows a slight decrease in skeletal radioactivity uptake.

The current study involved 2 Chinese Han families with early-onset severe PDB complicated by GCT. In family 1, the proband (Ⅲ-2), a 35-year-old man, experienced a left forearm fracture at the age of 26 years (Fig. 1A). Following the fracture, he progressively developed limb and skull deformities along with bone pain. He was diagnosed with severe polyostotic PDB, evidenced by multiple lytic lesions and deformities in the humeri and tibia as seen in the X-ray images (Fig. 1B). Bone scintigraphy showed increased tracer uptake in Paget lesions involving 21 bones (10.2%) of the skeletal system, including the skull, clavicle, spine, humeri, pelvis, femur, and tibia, as confirmed by correlative computed tomography, magnetic resonance imaging, and X-ray evaluations (Fig. 1C). By age 33 years, he developed tumors on the right top of the calva, which were subsequently removed through surgery and diagnosed as GCT (Fig. 1D). Subject II-2 (the proband's mother) fractured her left upper arm after an accidental fall at the age 35 years. She later developed multiple tumors in her lower jaw and died at the age of 39 years.

Family 2, comprising 4 members spanning 2 generations, has been previously described in terms of clinical features and medical history (Supplementary Fig. S1 (33)) (17). The existence of the L112P mutation within the *PFN1* gene of individual Ⅱ-2 in family 2 was confirmed using Sanger sequencing. Subsequently, the coding region of the *PFN1* gene in Ⅲ-2 of family 1 was sequenced, and a heterozygous missense mutation c.335T>C (NM_001375991.1) was identified in exon 3 of the *PFN1* gene. This mutation resulted in the substitution of leucine with proline at position 112 (Supplementary Fig. S2 (33)). The absence of this mutation in 500 healthy individuals implies that the *PFN1* missense mutation (L112P) might be the pathogenic mutation in this early-onset PDB/GCT family.

### Patients with PFN1 (L112P) Mutation Exhibited Limited Response to Zoledronate or Denosumab Treatment

The treatment and follow-up data records for all PDB patients carrying the *PFN1* (L112P) mutation, who were treated at the Department of Orthopedics Surgery at The Fourth Military Medical University-Affiliated Tangdu Hospital for more than 5 years, were compiled. The treatment of III-2 in family 1 was conducted in 2 stages. Initially, from 2013 to 2019, he received yearly doses of zoledronate (Aclasta 5 mg, Sandoz, Holzkirchen, Germany). However, normalization of serum ALP levels or relief of bone pain was not achieved during this period (Table 1). Subsequently, since the end of 2020, he has been receiving subcutaneous injections of denosumab (Xgeva 120 mg, Amgen) (once every 2 months). Despite a decrease in serum ALP levels, the level remained above normal, and there was no improvement in bone pain (Table 1). Ⅱ-2 in family 2 had been treated after diagnosis with denosumab (Xgeva 120 mg, Amgen) every 6 months to date. However, there had been no significant symptomatic relief, including bone pain, bone deformity, and headache. Bone scintigraphy performed on Ⅱ-2 in family 2 after 5 years of treatment indicated only a slight decrease in skeletal radioactivity uptake, suggesting limited effectiveness of denosumab on these patients (Fig. 1E).

**Table 1.** Treatment and follow-up of patients with *PFN1* (L112P) mutations

Subject	Follow-up times (y)	Treatment	Serum ALP (U/L)					Serum CTX (ng/L)					Serum P1NP (ng/mL)			
			Before therapy	1 y after therapy	2 y after therapy	5 y after therapy	7 y after therapy	Before therapy	1 y after therapy	2 y after therapy	5 y after therapy	7 y after therapy	Before therapy	1 y after therapy	3 y after therapy	5 yafter therapy
Ⅲ-2 of family 1	7.8	Zoledronate 5 mg (2013-2019) and Denosumab 120 mg (2019-2024)	2270	1720	1675	3517	1024	2227			2111	2074				>1200
Ⅱ-2 of family 2	5.3	Denosumab 120 mg	2124	697	831	503	139	3040	3240	2870	3809	3183			>1200	791.50

The normal range of serum ALP is 15 to 112 U/L. The normal range of serum CTX is 112 to 497 ng/L for females and 100 to 612 ng/L for males. The normal range of serum P1NP is 13.72 to 58.87 ng/mL for females and 16.89 to 65.49 ng/mL for male.

Abbreviations: ALP, alkaline phosphatase; CTX, C-terminal telopeptide of type 1 collagen; P1NP, procollagen type 1 N-terminal propeptide.

Briefly, both subjects exhibited markedly elevated serum ALP levels compared to patients with sporadic PDB undergoing similar treatment regimens. Moreover, markers of bone turnover, such as C-terminal telopeptide of type 1 collagen and procollagen type 1 N-terminal propeptide, did not exhibit significant improvement following treatment in these patients with PDB (Table 1). These findings suggested that inhibiting the RANKL-RANK pro-osteoclast differentiation pathway or bone resorption process may be insufficient to effectively treat the patients with PDB with *PFN1* (L112P) mutation, and that the mutation might show complex clinical consequences.

### Growth Failure and Skeletal Deformities in Mice with *Pfn1*^L112P^ Knock-in Mutation

**Figure 2.**
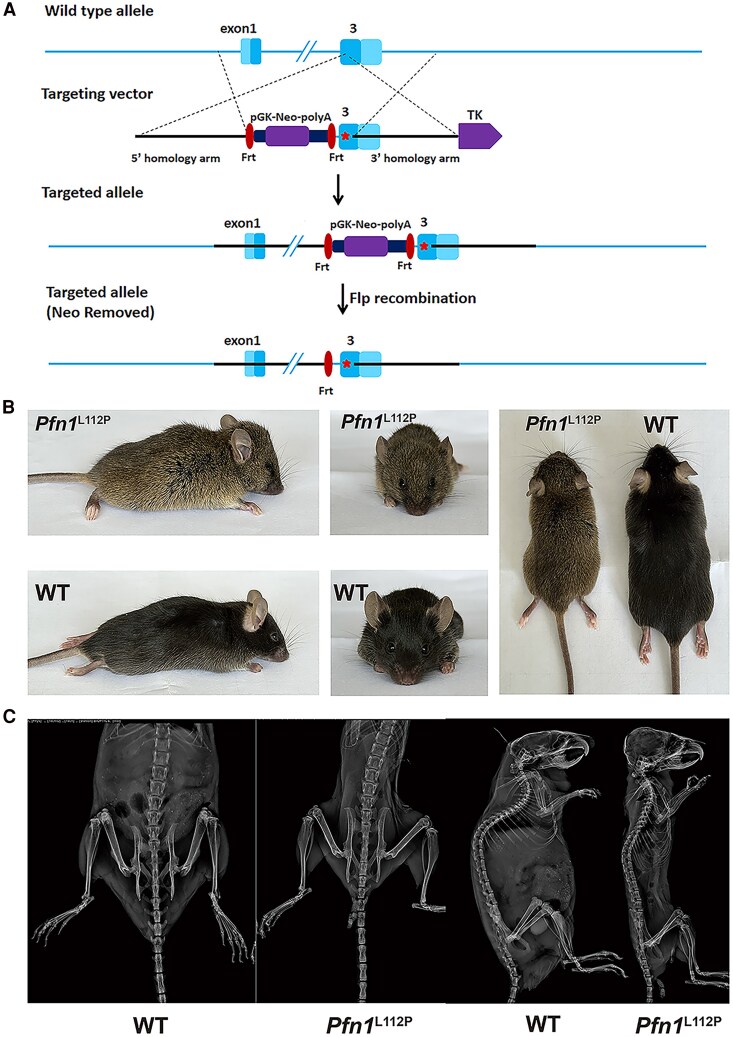
*Pfn1* knock-in mouse model and comparison of appearance between WT and *Pfn1*^L112P^ mice. (A) Targeting strategy for *Pfn1*^L112P^ knock-in mice. The knock-in resulted in a single amino acid substitution at position 112. (B) Appearance of WT and *Pfn1*^L112P^ mice. The altered facial appearance of *Pfn1*^L112P^ mice, compared to WT mice, was due to the wide-arched calva, shortened maxilla, and curved spine. This resulted in a steep nose-to-head curvature and arched body profile. (C) X-ray images of WT and *Pfn1*^L112P^ mice. Compared to WT mice, the *Pfn1*^L112P^ mice showed a thinner physique, cranial deformity, and a reduction in trabecular bone. All mice shown here were 8 weeks old and female.

A knock-in mouse model, *Pfn1*^L112P^, expressing the *Pfn1* L112P missense variant, was generated to explore the impact of *PFN1* missense mutation on PDB pathogenesis (Fig. 2A). Despite repeated mating attempts between heterozygous males and females, the homozygous mice could not be obtained, suggesting that homozygous *Pfn1* L112P mutation resulted in embryonic lethality. Conversely, heterozygous mutant mice exhibited PDB-like characteristics, including smaller size, widened skulls, broader skull sutures, and spinal curvature (Fig. 2B). Heterozygous mutant mice were thereafter referred to as *Pfn1*^L112P^ mice. X-ray imaging of heterozygous mice revealed cranial deformities, spinal curvature, and reduced trabecular bone density in the skeletal structure (Fig. 2C).

**Figure 3.**
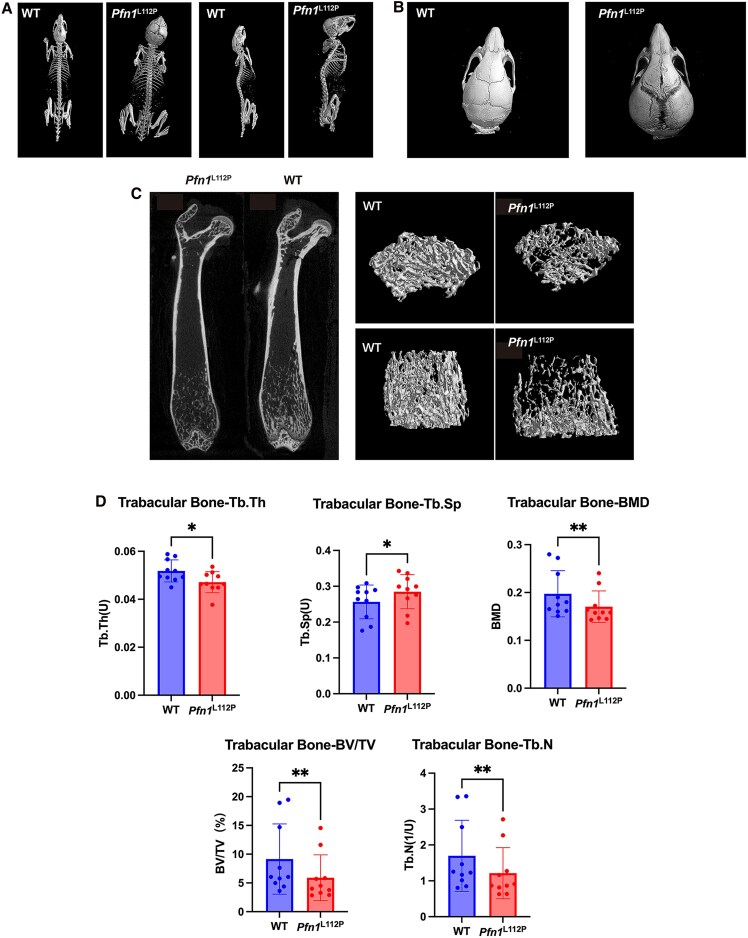
Comparison of morphology and 3-dimensional reconstruction between WT and *Pfn1*^L112P^ mice by micro-CT. (A) Appearance of WT and *Pfn1*^L112P^ mice. The *Pfn1*^L112P^ mice showed broad arched calvaria, shortened maxilla, and curved spine *Pfn1*^L112P^ mice compared to WT mice. (B) Skull 3-dimensional reconstruction between WT and *Pfn1*^L112P^ mice. The *Pfn1*^L112P^ mice showed cranial deformity with enlarged cranial sutures compared to WT mice. (C) Images of 3-dimensional reconstruction of the femurs in WT and *Pfn1*^L112P^ mice. All mice shown here were 8 weeks old and female. (D) Comparison of the levels of Tb.Th, Tb-BV/TV, Tb.Sp, Tb.N, and Tb-BMD between WT and *Pfn1*^L112P^ mice (n = 10 for each group; *P* = .0064 for Tb.N, *P* = .0093 for Tb-BV/TV, *P* = .0084 for Tb-BMD, *P* = .0156 for Tb.Sp, and *P* = .0199 for Tb.Th.) All data were analyzed using Student *t*-test.

The 3-dimensional micro-computed tomography reconstruction analysis of the entire skeleton was used, and morphometric assessments on the trabecular and cortical femurs of 8-week-old mice were conducted to examine skull deformities with greater precision and objectivity. The 3-dimensional reconstruction of *Pfn1*^L112P^ mice revealed a wide cranial suture and deformed craniofacial bones and spines compared to WT mice (Fig. 3A and 3B).

### Decreased Bone Mass and Damaged Microstructure of Trabecular Bone in *Pfn1*^L112P^ Mice

The micro-computed tomography analysis also revealed that the heterozygous mutant mice exhibited lower trabecular bone density compared to WT mice. The mutant mice showed significantly reduced trabecular bone volume fraction, trabecular thickness, trabecular number, and bone mineral density and increased trabecular separation compared to WT mice (Fig. 3C and 3D). The active bone resorption in the trabecular bone is consistent with the PDB phenotype.

### *Pfn1* ^L112P^ Enhances Osteoclast Differentiation and Resulted in the Formation of Larger Osteoclasts

**Figure 4.**
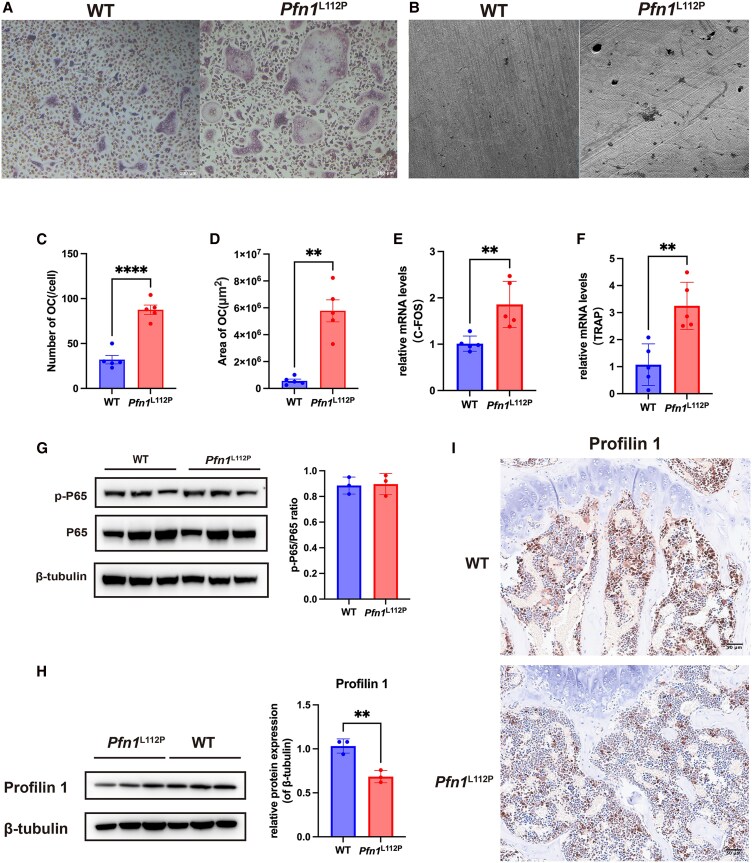
*PFN1* ^L112P^ mutation results in enhanced osteoclast differentiation. (A) TRAP staining of osteoclasts derived from WT and *Pfn1*^L112P^ mice. (B) Electron microscopy images of the resorption pits of WT and *Pfn1*^L112P^ osteoclasts cultured on bone slices. (C) Comparison of the numbers of TRAP-positive osteoclasts (*P* < .0001; n = 5 for each group). (D) Comparison of the area of TRAP-positive osteoclasts (*P* = .0079; n = 5 for each group). (E) Comparison of the relative mRNA levels of *C-FOS* between WT and *Pfn1*^L112P^ mice (*P* = .0070; n = 5 for each group). (F) Comparison of the relative mRNA levels of TRAP between WT and *Pfn1*^L112P^ mice (*P* = .0031; n = 5 for each group). (G) Western blot analysis of p-P65 and P65 from osteoclast cultures between WT and *Pfn1*^L112P^ mice (*P* > .05; n = 3 for each group). (H) Western blot analysis of Profilin 1 from osteoclast cultures between WT and *Pfn1*^L112P^ mice (*P* = .0048; n = 3 for each group). (I) Immunohistochemistry staining showed a decrease in Profilin 1 expression in bone marrow-mesenchymal stem cells from *Pfn1*^L112P^ mice compared with WT mice. All data were analyzed using Student *t*-test.

**Figure 5.**
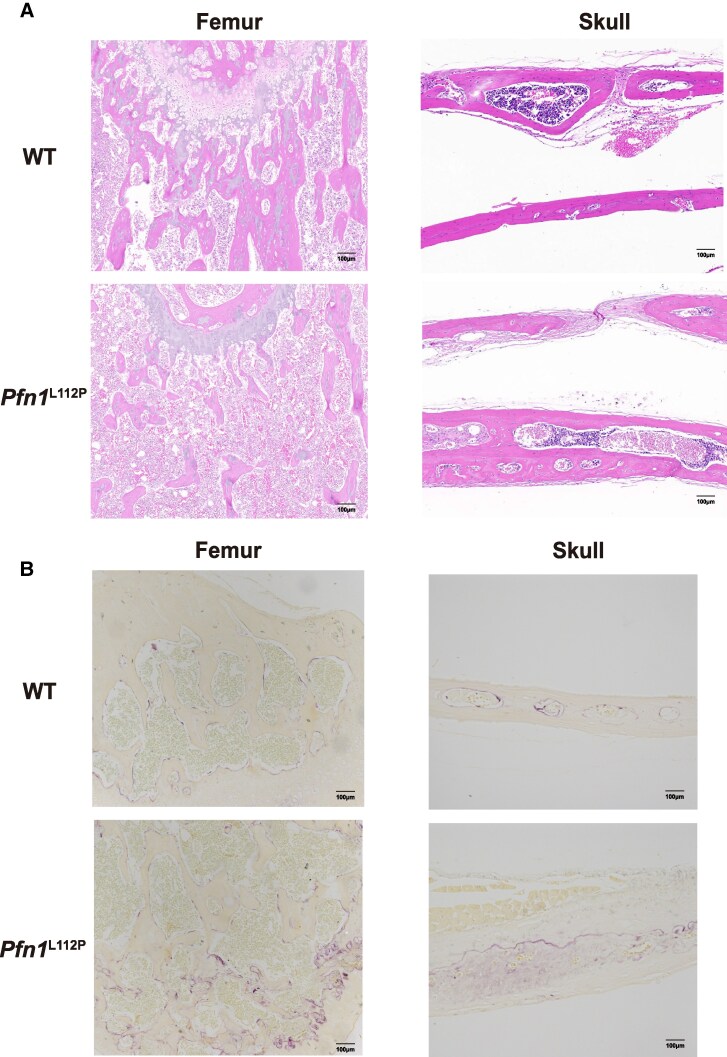
Hematoxylin and eosin (H&E) staining and TRAP staining of the distal femur and skull sections from WT and *Pfn1*^L112P^ mice. (A) H&E staining of femur sections showed reduced and disordered trabeculae in *Pfn1*^L112P^ mice compared to WT mice. H&E staining of skull sections showed widened, deformed, and degraded skull sections in *Pfn1*^L112P^ mice compared to WT mice. (B) TRAP staining revealed a notable increase in multinucleated osteoclasts located on the trabecular surfaces of the femurs in *Pfn1*^L112P^ mice. TRAP staining revealed a notable increase in multinucleated osteoclasts located on the cavity surfaces of the skull in *Pfn1*^L112P^ mice.

An aberrant differentiation of osteoclasts is a hallmark feature of PDB. The bone marrow macrophages (BMMs) from the femurs of 2-month-old *Pfn1*^L112P^ mutant mice and control mice were isolated to examine the function of *Pfn1*^L112P^ mutation on osteoclastogenesis. These culture cells were then induced to undergo osteoclast differentiation. TRAP staining showed an increased number and size of osteoclasts in BMM cultures from *Pfn1*^L112P^ mice (Fig. 4A, 4C, and 4D). In addition, the resorption pit formation assay revealed that osteoclasts derived from *Pfn1*^L112P^ mice tended to resorb more mineralized matrix by larger and deeper pits (Fig. 4B). Furthermore, qPCR results showed elevated expression levels of osteoclast differentiation and resorption-related markers, such as c-FOS and TRAP in BMM cultures from *Pfn1*^L112P^ mice (Fig. 4E and 4F). These findings suggested that the *Pfn1*^L112P^ mutation promoted the differentiation of mononuclear cells into osteoclasts. We also compared the activation of NF-kB in these cell cultures by Western blot. It was found that heterozygous mutation of *Pfn1* did not affect NF-κB activation in osteoclast cultures (Fig. 4G). Subsequently, the expression levels of Profilin 1 in these cell cultures were assessed through Western blot, revealing a modest reduction in the protein expression in osteoclast cultures derived from *Pfn1*^L112P^ mice (Fig. 4H). We next demonstrated expression of Profilin 1 in femur sections using immunohistochemistry staining. The results showed a significant decrease in expression in mesenchymal stem cells (MSCs) from *Pfn1*^L112P^ mice compared with WT mice (Fig. 5I). These findings are consistent with our prediction of structural modeling and thermodynamic analysis of the mutant Profilin 1 (L112P).

A comparative analysis of frontal sections from the distal femurs and skulls of 8-week-old mice using hematoxylin and eosin staining was conducted to explore variations in bone histology. The reduction in the number and disarray of trabeculae in *Pfn1*^L112P^ mice was observed as compared to their WT counterparts (Fig. 5A). Moreover, the skulls of *Pfn1*^L112P^ mice showed increased thickness and widened skull sutures. The skull bone tissue displayed extensive degradation attributed to hyperactive osteoclasts, indicative of characteristic focal osteolytic lesions (Fig. 5A). Moreover, TRAP staining demonstrated a significant presence of multinucleated TRAP-positive cells in the endosteal regions spanning from the metaphysis to the diaphysis in *Pfn1*^L112P^ mice, whereas osteoclast distribution was typically scarce in WT mice (Fig. 5B). To investigate the osteoblast differentiation, we isolated bone marrow-derived MSCs from 8-week-old WT and *Pfn1*^L112P^ mice, which were then subjected to osteoblast differentiation. However, ALP staining, Alizarin Red Staining, and qPCR results of osteoblast differentiation markers shows no difference between WT and *Pfn1*^L112P^ mice (Supplementary Fig. S3A-B (33)), indicating that *PFN1* L112P mutation primarily influences osteoclast function rather than osteoblast differentiation.

### Increased Actin-ring Like Structures at the Bone Surface in *Pfn1*^L112P^ Mice

**Figure 6.**
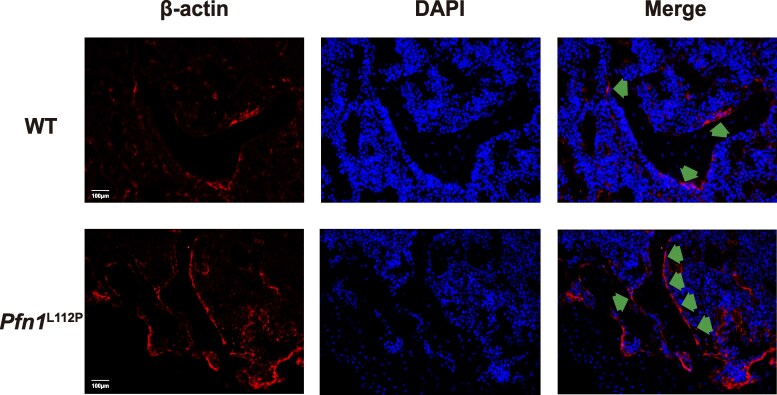
*PFN1* ^L112P^ mutation affects the acting-ring structure at the bone surface. *Pfn1*^L112P^ mice femur sections revealed an increase in the abundance of actin-ring like structures (indicated with arrows) at the bone surface compared to WT mice (200×).

Profilin 1 directly associates with actin. We investigated the impact of the *Pfn1* L112P mutation on the actin-ring structure in osteoclasts by conducting β-actin immunofluorescence staining on femur sections, as previously reported (34). The findings indicate that *Pfn1*^L112P^ mice exhibited a significantly higher number of actin-ring structures on the bone surface compared to WT mice (Fig. 6), likely due to the activation of osteoclasts and the enhanced formation of actin rings driven by the *Pfn1* L112P mutation.

### *Pfn1* ^L112P^ Mice Exhibited No Sign of Muscle Weakness

The rotarod test was conducted on *Pfn1*^L112P^ mice and their WT counterparts from 12 to 14 weeks of age to explore the impact of the *Pfn1* L112P mutation on the function of motor neurons in mice. No significant difference in rotarod performance between the mutant and control group mice was observed (Supplementary Table S1 (33)). Moreover, manifestations of hind limb weakness or abnormal gait were not observed either (Supplementary Table S1 (33)). Additionally, the body weight and of the *Pfn1*^L112P^ and WT mice did not differ significantly at either time point (Supplementary Table S2 (33)).

## Discussion

**Figure 7.**
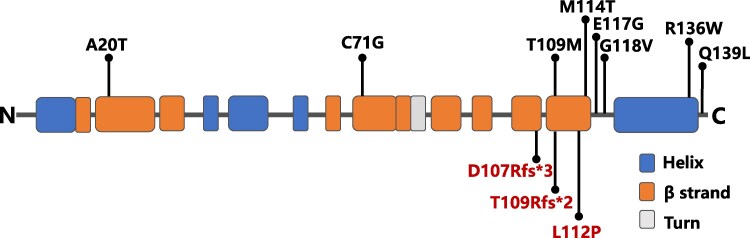
Profilin 1 protein structure and locations of PFN1 variants in patients with amyotrophic lateral sclerosis (ALS) and Paget disease of bone (PDB). PFN1 is characterized by 5 α-helices and 7 β-sheets. The ALS variants are depicted above the protein structure, and the PDB variant is depicted below the protein structure with a red color.

Previous studies reported that all *PFN1* missense mutations, including A20T, C71G, T109M, M114T, E117G, G118V, R136W, and Q139L (35), were associated with ALS (Fig. 7A). The current study identified a *PFN1* missense mutation c.335T>C (L112P) in another early-onset PDB pedigree, a mutation that was not previously reported in ALS cases. The comprehensive interviews and physical examinations did not show any ALS-related symptoms or signs in PDB patients carrying the *PFN1* L112P mutation. Moreover, none of the family members with this mutation had ALS. Additionally, a *Pfn1*^L112P^ knock-in mouse model was successfully generated, and the results showed that the mutated mice exhibited PDB-like characteristics, including abnormal growth and development, changes in skeletal morphology, reduced trabeculae in bones, and upregulation of osteoclast function and deregulation of osteoblast function. However, the mutant mice showed normal rotarod performance, hind limb strength, and normal gait, which were the indicators of motor neuron defects, indicating that the *PFN1* missense mutation L112P caused early-onset PDB but not ALS in mice. Notably, the L112P mutation was in close proximity to ALS-related *PFN1* missense mutations, including T109M, M114T, E117G, and G118V. Nevertheless, both the human and mouse data showed that these 5 mutations are located with a 10-amino-acid stretch in PFN1 and can cause rather distinct phenotypes in humans and mice.

Previous studies showed that knock-down of *PFN1* enhanced the migration and bone resorption abilities of RAW264.7 osteoclast precursor cells (36). While the deletion of *Pfn1* caused embryonic lethality, conditional knockout of *Pfn1* in monocytes/osteoclasts increased the migration and bone resorption capabilities of osteoclasts with minimal impact on their size and number (36). Studies on *PFN1* mutations in ALS pathogenesis have attempted to explore the mechanistic effect of mutations in *Pfn1* on osteoclast migration and function. Previous studies have reported that ALS pathogenesis is associated with TDP-43 accumulation in motor neurons, where it acts as a coactivator of the NF-κB p65 subunit, thereby enhancing NF-κB signaling pathway activation (28). Recent investigations also support the notion that *PFN1* plays a negative role in inhibiting NF-κB activation (30, 31). Considering that the NF-κB signaling pathway is vital for osteoclast proliferation and differentiation as well as bone resorption function, it was speculated that the *PFN1* L112P mutation could enhance osteoclast differentiation and function by promoting NF-κB pathway activation. However, we found that heterozygous mutation of *Pfn1* does not affect NF-κB activation in osteoclast cultures, thus excluding a possible role for *PFN1* L112P mutation in the RNAKL-RANK-NF-κB pathway. Consequently, neither patients nor mice display ALS phenotypes. This is consistent with the observations that giant osteoclasts were formed in *Pfn1* L112P mutation cultures and that PDB patient carrying *PFN1* L112P mutation are refractory to the treatment with anti-RANKL antibody or bisphosphonate.

Profilin 1, an actin-binding protein, is essential for cytoskeletal formation and reorganization (37-39). Actin forms an “actin-ring” structure crucial for osteoclast-mediated bone resorption (40). This ring-like structure positions itself at the osteoclast bone interface, establishing a “sealing zone” that merges the cell membrane with the bone surface. This arrangement creates an acidic environment conducive to bone resorption. In our study, *Pfn1*^L112P^ mouse femur sections showed an increase in the number of actin ring-like structures at the bone surface; therefore, it was speculated that the *Pfn1* L112P mutation might affect the formation of the “actin ring,” impacting the bone resorption process.

We summarized the clinical manifestations of all PDB patients with *PFN1* mutations (Table 2). Our Chinese patients experience malignant transformation in their 30s during an active disease phase characterized by prominent monocyte/osteoclast activity, potentially predisposing to GCT formation. In contrast, the osteosarcoma transformation in the Italian patients may relate to their later disease onset (>40 years) (15, 16), where PDB was likely in the quiescent/sclerotic phase with compensatory osteoblast hyperactivity favoring osteosarcoma development. These differences may further reflect variations in genetic background, environmental influences, and epigenetic factors.

**Table 2.** Summary of clinical manifestations in reported patients with PDB with *PFN1* mutations

Reference	Merlotti et al (15)	Scotto et al (16)	Wei et al (17)	The present study
Total cases	5	11	11	1
Country	Italy	Italy	China	China
Sex ratio (F/M)	5/0	8/3	6/5	0/1
DNA change	p. D107Rfs*3	p. D107Rfs*3	p. D107Rfs*3 p. L112P p. T109Rfs*2	p. L112P
Age (y), mean ± SD				
Onset	33 ± 8	32 ± 9	31 ± 8	26
Diagnosis	34 ± 10	NM	44 ± 16	32
Affected sites (n), mean ± SD	8 ± 3	5.8 ± 2	NE	6
ALP at diagnosis (U/L), *M* (*P*_25_, *P*_75_)	839 (726 940)	NM	275 (204, 406)	2270
Tumor				
Onset (y)	NM	47 ± 7	32 ± 7	33
OS/GCT	1/0	3/0	0/4	0/2
Site	Humerus	Scapula	Ilium/nasal cavity/forehead	Calva
PDB symptoms (affected/unaffected)				
Bone pain	5/0	NM	4/7	Yes
Headache	5/0	NM	6/5	Yes
Bone deformity	NM	NM	3/8	Yes
Hearing loss	3/2	NM	3/8	No
Fracture	2/3	6/5	3/8	Yes
Osteoarthritis	5/0	NM	NM	Yes
Treatment				
Bisphosphonate/deno-sumab/calcitonin	5/0/0	0/0/3	7/1/0	Bisphosp-honate and denosumb
Symptom relief (yes/no)	0/5	0/3	2/6	No

Symptom relief means achieving the normalization of alkaline phosphatase levels or the remission of pain.

Abbreviations: GCT, giant cell tumor; NE, not evaluated; NM, not mentioned; OS, osteosarcoma.

In patients with sporadic PDB, treatment with bisphosphonates (41, 42) (zoledronate 5 mg once yearly) or denosumab (43, 44) (60 mg once every 6 months) normalized serum ALP levels and substantially relieved clinical symptoms, such as bone pain. However, patients carrying the *PFN1* L112P mutation exhibited a poor response to zoledronate or denosumab compared to sporadic cases, consistent with the observations that *PFN1* L112P mutation does not affect the RANKL-RANK pro-osteoclast differentiation pathway, suggesting that inhibiting bone resorption process alone is insufficient to effectively treat this early-onset and more severe PDB. The decrease in osteoblast function is also a contributing factor to the limited effectiveness of denosumab. However, further investigation is required to confirm this hypothesis.

Briefly, the current study conducted on both human and mouse subjects indicated that the *PFN1* missense mutation (L112P) caused the early-onset PDB, likely by impacting the actin-mediated bone resorption process. The severity of osteoclast and bone resorption phenotypes surpassed those observed in osteoclast-specific *Pfn1*-knockout mice, indicating a dominant negative effect of this *PFN1* mutant variant. Despite its proximity to ALS-linked mutations, the L112P mutation induced PDB and not ALS in humans or mice, suggesting distinct effects on the interaction with effector molecules. The *Pfn1*^L112P^ mouse model generated in the current study holds promise for elucidating the pathological mechanisms underlying PDB. Furthermore, the current study provides an explanation for the resistance of the PDB patients harboring the *PFN1* L112P mutation to the treatment with bisphosphonate or RANKL antibodies.

## Data Availability

All data generated or analyzed during this study are included in this published article and its supplementary files.

## Abbreviations

ALP: alkaline phosphatase
ALS: amyotrophic lateral sclerosis
BMM: bone marrow macrophage
ES: embryonic stem
GCT: giant cell tumor
MSC: mesenchymal stem cell
NF-κB: nuclear factor-κB
PDB: Paget disease of bone
PFA: paraformaldehyde
qPCR: quantitative PCR
RT: room temperature
TRAP: tartrate-resistant acid phosphatase
WT: wild-type
